# Detecting Colorectal Adenomas and Cancer Using Volatile Organic Compounds in Exhaled Breath: A Proof-of-Principle Study to Improve Screening

**DOI:** 10.14309/ctg.0000000000000518

**Published:** 2022-08-18

**Authors:** Hao Ran Cheng, Robert W.R. van Vorstenbosch, Daniëlle M. Pachen, Lonne W.T. Meulen, Jan Willem A. Straathof, Jan W. Dallinga, Daisy M.A.E. Jonkers, Ad A.M. Masclee, Frederik-Jan van Schooten, Zlatan Mujagic, Agnieszka Smolinska

**Affiliations:** 1 Division of Gastroenterology-Hepatology, Department of Internal Medicine, Maastricht University Medical Center+, Maastricht, the Netherlands;; 2 Department of Gastroenterology and Hepatology, Máxima Medical Center, Veldhoven, the Netherlands;; 3 GROW, School for Oncology and Reproduction, Maastricht University, Maastricht, the Netherlands;; 4 NUTRIM, School of Nutrition & Translational Research in Metabolism, Maastricht University, Maastricht, the Netherlands;; 5 Department of Pharmacology and Toxicology, Faculty of Health, Medicine and Life Sciences, Maastricht University, Maastricht, the Netherlands.

## Abstract

**INTRODUCTION:**

Early detection of colorectal cancer (CRC) by screening programs is crucial because survival rates worsen at advanced stages. However, the currently used screening method, the fecal immunochemical test (FIT), suffers from a high number of false-positives and is insensitive for detecting advanced adenomas (AAs), resulting in false-negatives for these premalignant lesions. Therefore, more accurate, noninvasive screening tools are needed. In this study, the utility of analyzing volatile organic compounds (VOCs) in exhaled breath in a FIT-positive population to detect the presence of colorectal neoplasia was studied.

**METHODS:**

In this multicenter prospective study, breath samples were collected from 382 FIT-positive patients with subsequent colonoscopy participating in the national Dutch bowel screening program (n = 84 negative controls, n = 130 non-AAs, n = 138 AAs, and n = 30 CRCs). Precolonoscopy exhaled VOCs were analyzed using thermal desorption-gas chromatography-mass spectrometry, and the data were preprocessed and analyzed using machine learning techniques.

**RESULTS:**

Using 10 discriminatory VOCs, AAs could be distinguished from negative controls with a sensitivity and specificity of 79% and 70%, respectively. Based on this biomarker profile, CRC and AA combined could be discriminated from controls with a sensitivity and specificity of 77% and 70%, respectively, and CRC alone could be discriminated from controls with a sensitivity and specificity of 80% and 70%, respectively. Moreover, the feasibility to discriminate non-AAs from controls and AAs was shown.

**DISCUSSION:**

VOCs in exhaled breath can detect the presence of AAs and CRC in a CRC screening population and may improve CRC screening in the future.

## INTRODUCTION

Colorectal cancer (CRC) is the third most common cancer worldwide and poses an important healthcare issue with significant morbidity, mortality, and economic impact (1). CRC is known to develop from precursor lesions, in most cases adenomas, through the adenoma-carcinoma sequence (2–4). CRC can be prevented if these precursor lesions are identified and removed endoscopically (5). Therefore, worldwide bowel cancer screening programs have been implemented, which significantly reduced CRC-related morbidity and mortality (6,7). In most countries, including the United States and many European countries, bowel cancer screening programs are based on the fecal immunochemical test (FIT) for hemoglobin, followed by a colonoscopy if the FIT is positive (8). However, this screening approach has significant limitations. FIT suffers from a considerable number of false-positives and false-negatives (9,10). At a specificity of 95%, the sensitivity of FIT is 73% for CRC and as low as 25% for advanced adenomas (AAs) (11). Moreover, CRC, AAs, and non‐advanced adenomas (NAAs) were found in only 7%, 39%, and 23%, respectively, of all colonoscopies performed after a positive FIT (9). In other words, positive FITs are often followed up by an unnecessary colonoscopy bringing a high burden to the healthcare system and society while a large proportion of relevant endoscopic findings (i.e., CRC and especially AA) are being missed. These limitations may be overcome by the implementation of a noninvasive and promising approach using volatile organic compounds (VOCs) in exhaled breath to detect not only CRC but also its precursor lesions in the FIT-positive population.

VOCs consist of a large variety of endogenous and exogenous metabolites originating from the host and microbial metabolism. They are measurable in exhaled breath, feces, blood, urine, and saliva (12,13). Alterations in the host and microbiome metabolism related to colorectal neoplasia are reflected in exhaled breath VOC profiles (14,15). Earlier studies have analyzed exhaled VOCs mostly in patients with CRC (16–21). However, translation to clinical practice is hampered by the lack of study designs that compare relevant biological variation (healthy vs early CRC and adenomas). In addition, inappropriate consideration of potential confounding effects (e.g., bowel cleansing, age) results in bias of reported outcomes. As a result, although the data on VOCs for CRC detection are promising so far, implementation in clinical practice is not yet in sight (22–24).

Therefore, this study considered only FIT-positive individuals to reflect the true relevant variation in the general population while minimizing sampling bias. The aim of this multicenter prospective study was to assess the feasibility of exhaled breath analysis to differentiate between CRC, AA, NAA, and negative controls in a FIT-positive CRC screening population.

## METHODS

### Study design and population

In the national Dutch bowel screening program, all inhabitants between 55 and 75 years are biennially invited to participate by providing a FIT sample. After a positive FIT, subjects are invited for a prescreening intake for colonoscopy. Subjects scheduled for this outpatient clinic visit in the Máxima Medical Center (Veldhoven, the Netherlands) and the Maastricht University Medical Center+ (Maastricht, the Netherlands) between July 2016 and January 2018 were invited to participate in this study. Breath samples were collected before bowel cleansing for colonoscopy and after written informed consent had been obtained. Only patients undergoing a scheduled colonoscopy were included in this study. All colonoscopies were performed by certified endoscopists, minimalizing chances of missed lesions (25). The Boston Bowel Preparation Scale was used to assess cleanliness of the bowel for adequate inspection (26). Patients with inflammatory bowel disease, familial polyposis syndromes, or active malignancies other than CRC; those under current treatment with radiotherapy or chemotherapy; or those unable or unwilling to provide informed consent were excluded. AA was defined as size ≥ 1 cm, villous histology, and/or high-grade dysplasia. Based on the endoscopic findings, patients were categorized into 4 groups: CRC, AA, NAA, and negative controls. In the case of multiple lesions, the classification was based on the most advanced lesion found. This study was approved by the Medical Ethics Research Committee of the Maastricht University Medical Center+ (METC No. 16-4-103.1/ab).

### Breath sampling procedure and analysis

Patients were asked to inflate a 3-L Tedlar bag (SKC Ltd., Dorset, UK). Within an hour of collection, its contents were transferred into carbon-filled stainless-steel desorption tubes (Markes International, Llantrisant Business Park, UK). In each study center, 1 room was assigned to be used for breath collection purposes. Breath samples were analyzed by thermal desorption-gas chromatography coupled with time-of-flight mass spectrometry (TD-GC-MS), as described previously (27).

### Baseline statistical analysis

Clinical, anthropometric, demographic, endoscopic, and histopathology data were collected in a standardized manner as part of the national Dutch bowel screening program using hospital records. These included age, sex, body mass index (BMI), smoking, alcohol use, medication use, medical history, endoscopic findings, and histopathology.

IBM SPSS statistical software (version 22.0; IBM, Armonk, NY) was used for statistical analysis of the baseline patient demographics. Age, BMI, medication use, medical history, endoscopic findings, and histopathology are presented as mean with corresponding SD, median with corresponding interquartile range, or a fixed number with relative percentage. Differences in baseline characteristics were tested using the χ^2^ test (dichotomous data), and 1-way analysis of variance was performed to compare differences between means in 2 or more groups. A 2-sided α-level < 0.05 was defined as statistically significant.

### Breath data preprocessing

TD-GC-MS data were preprocessed before subsequent statistical analysis. This consisted of noise removal, baseline correction, alignment, normalization, peak picking, and scaling, as previously described (see Supplementary Table 1, Supplementary Digital Content 1, http://links.lww.com/CTG/A853, which summarizes the breath data preprocessing in detail) (28). Only features detected in at least 20% of 1 of the categorized disease classes (i.e., CRC, AA, NAA, and negative controls) were included in the analysis. The data were corrected for instrumental variation using ComBat and Surrogate Variable Analysis (29–32). Putative identification of VOCs was performed using the National Institute of Standards and Technology library. All breath data analyses were performed using MATLAB 2018a—Statistics and Machine Learning Toolbox.

### Breath data analysis

Three supervised machine learning models were created to predict endoscopic findings. Model 1 distinguished CRC cases from negative controls. Model 2 and Model 3 discriminated AA against negative controls (2-class classification) and AA vs NAA vs negative controls (3-class classification), respectively.

#### Model 1: discriminating CRC vs negative controls.

The number of CRC cases was relatively low, that is, 30, resulting in class imbalanced data with low statistical power that negatively influence the reliability of discriminatory models and biomarker selection. To circumvent biased biomarker selection, 1-class classification was applied using the Isolation Forest algorithm (which does not require biomarker selection) with leave-one-out cross validation (33). The overall procedure is shown in the supplementary material (see Supplementary Text, Supplementary Digital Content 2, http://links.lww.com/CTG/A854, which describes a more elaborate explanation and description of the statistical procedure).

#### Model 2: discriminating AA vs negative controls and AA plus CRC combined vs controls.

**Figure 1.**
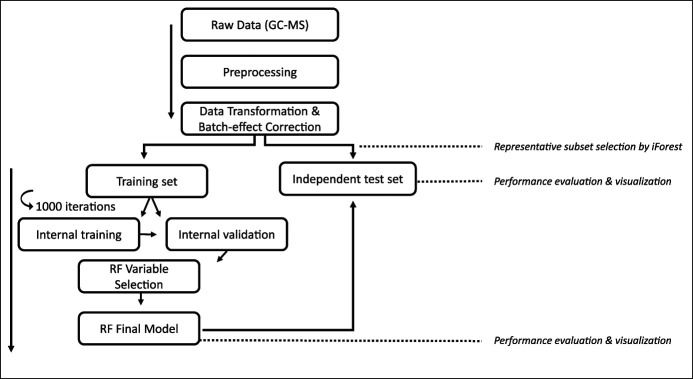
Representation of the data analytics that were applied in this study to discriminate advanced adenomas from negative controls. In this study, ComBat was applied as a batch effect correction technique and Isolation Forests were used to select the representative subset for the independent test set. GC-MS, gas chromatography–mass spectrometry; RF, Random Forest.

To discriminate AA from negative controls, Random Forest (RF) was used (called here Model 2). The data set was first split into a training set (n = 128 AAs, n = 74 controls) and independent internal test set (n = 10 AA, n = 10 controls) using Isolation Forest (33). Variable selection and optimization was based on the variable importance as assessed by an internal iterative validation procedure of RF (1,000 iterations with 1,000 trees per iteration ) (34). Next, the final model was tested on the independent internal test set. A stepwise overview of the procedure is presented in Figure 1. Using Principal Coordinate Analysis (PCoA) on the out-of-bag proximities obtained by RF, the data were visualized (see Supplementary Text, Supplementary Digital Content 2, http://links.lww.com/CTG/A854, which describes this procedure in further detail). The performance of Model 2 was evaluated using sensitivity, specificity, area under the curve, receiver operating characteristic (ROC) curves, positive predictive value (PPV), and precision-recall curves (35). Because Model 1 contained insufficient data for biomarker selection, another RF model was created based on the selected VOCs by Model 2 to assess the applicability and performance of this model to discriminate CRC from negative controls and CRC and AA combined from negative controls.

#### Model 3: discriminating AA, NAA, and negative controls.

**Figure 2.**
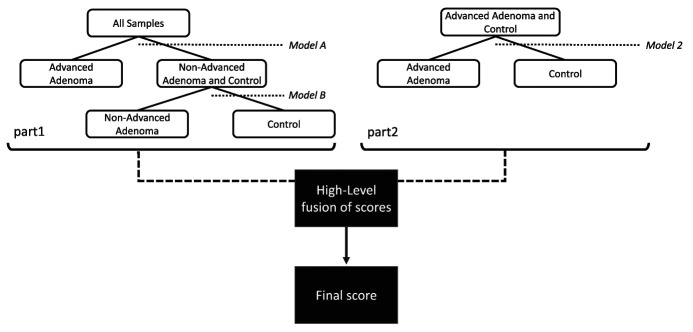
Hierarchical modeling approach to discriminate between negative controls, AA, and NAA using 3 sequential binary Random Forest models. First, AA was discriminated against the combination of controls and NAA. Subsequently, the latter were discriminated. To add an extra layer of sensitivity, Model 2 was added. AA, advanced adenoma; NAA, nonadvanced adenoma.

In Model 3, a 3-class classification model was built to discriminate between AA, NAA, and controls using RF. The procedure used here is shown in Figure 2. First, Model A (discriminating AA vs rest) and Model B (discriminating NAA vs controls) were created. Here, internal training (n = 123 AA, n = 115 NAA, n = 69 controls) and internal validation (n = 15 AA, n = 15 NAA, n = 15 controls) sets were used to optimize the model. Using the final obtained models, the PCoA scores based on the RF proximity matrices were assessed on separating the classes of interest. This resulted in the selection of the scores of the first Principal Coordinates (PCos) for all models. Subsequently, these scores (Model A and Model B) were combined in a hierarchical fashion, as illustrated in Figure 2, part 1. Similarly, the first PCo scores of Model 2 were calculated and combined with the subset obtained in Models A and B (Figure 2, part 2) midst a midlevel fusion approach. Finally, using all obtained PCoA scores, a final 3-class model was built, visualized, and assessed using weighted accuracy.

## RESULTS

### Baseline characteristics

**Figure 3.**
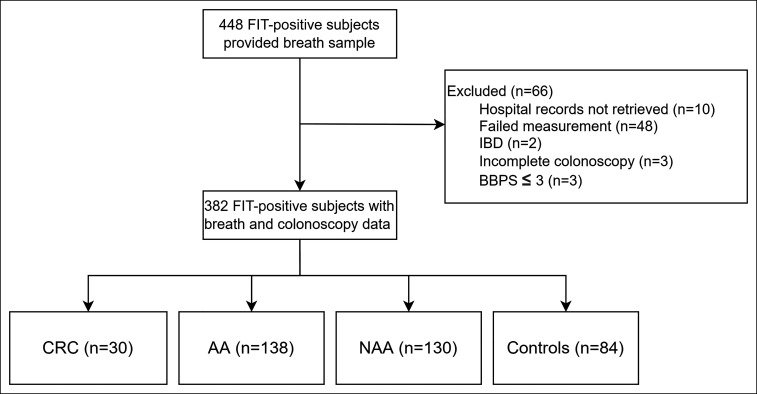
Flowchart of included subjects. AA, advanced adenoma; BBPS, Boston Bowel Preparation Scale; CRC, colorectal cancer; FIT, fecal immunochemical test; IBD, inflammatory bowel disease; NAA, nonadvanced adenoma.

Four hundred forty-eight patients participated in this study and provided a breath sample as shown in Figure 3. Hospital records of 10 patients (4 MMC, 6 MUMC+) could not be retrieved within the hospitals' medical data system and were excluded from this study. Three patients had a Boston Bowel Preparation Scale of 3, and 2 patients had a history of IBD and were excluded from this study. Three patients had an incomplete colonoscopy because of benign stenosis, diverticulitis, or technical aspects of the procedure. Forty-eight breath samples contained failed measurements because of low sensitivity in the recorded mass spectra. Three hundred eighty-two patients with breath, colonoscopy, and histopathology data were included for analysis. Thirty patients (7.9%) had CRC; 138 patients (36.1%) had AA; 130 patients (34%) had NAA; and 84 patients (22%) were negative for CRC, AA, and NAA and comprised the control group.

Baseline characteristics are summarized in Table 1, and endoscopic findings and medication usage are listed in Table 2. Age was comparable between all groups. Smoking status and alcohol usage was comparable between all groups, although data were partially missing. Patients in the AA group were more frequently of male sex compared with CRC (*P* = 0.032) and controls (*P* = 0.001), had higher BMI compared with controls (*P* = 0.026), and were more likely to have hypertension (*P* = 0.045). Patients in the NAA group more frequently used acetylsalicylic acid compared with the AA group (*P* = 0.02), and nonsteroidal anti-inflammatory drugs were more often used by controls compared with all other groups.

**Table 1.** Baseline characteristics

Baseline characteristic	CRC (n = 30)	AA (n = 138)	NAA (n = 130)	Controls (n = 84)	*P* value
Age, yr ± SD	66.5 (5.7)	64.4 (7.4)	65.4 (4.9)	64.8 (8.6)	NS
Male, n (%)	14 (46.7)	93 (67.4)	76 (58.5)	37 (44)	^a^
BMI, kg/m^2^ ± SD	27.8 (5.6)	28 (4.8)	27.2 (4.7)	27.2 (4.7)	^b^
Smoking, n (%)	3 (10)	22 (15.9)	15 (11.5)	8 (9.5)	NS
No	9 (30)	65 (47.1)	54 (41.5)	34 (40.5)	
Unknown	18 (60)	51 (37)	61 (47)	42 (50)	
Alcohol, n (%)	5 (16.7)	48 (34.8)	40 (30.7)	24 (28.6)	NS
No	7 (23.3)	37 (26.8)	29 (22.3)	18 (21.4)	
Unknown	18 (60)	53 (38.4)	61 (47)	42 (50)	
CRC TNM stadium, n (%)					
T1N0M0	12 (40)				
T2N0M0	7 (23.3)				
T3N0M0	3 (10)				
T1N1M0	1 (3.3)				
T2N1M0	3 (10)				
T3N1M0	4 (13.3)				

AA, advanced adenoma; BBPS, Boston Bowel Preparation Scale; BMI, body mass index; CRC, colorectal cancer; NAA, nonadvanced adenoma; NS, not significant.

a CRC vs AA (*P* = 0.032), AA vs controls (*P* = 0.001), NAA vs controls (*P* = 0.039).

b AA vs controls (*P* = 0.026).

**Table 2.** Endoscopic findings and medication usage

Endoscopic finding	CRC (n = 30)	AA (n = 138)	NAA (n = 130)	Controls (n = 84)	*P* value
Diverticulosis, n (%)	7 (23.3)	65 (47.1)	68 (52.3)	39 (46.4)	a
Diverticulitis, n (%)	0 (0)	3 (2.2)	1 (0.8)	1 (1.2)	NS
Angiodysplasia, n (%)	1 (3.3)	1 (0.7)	5 (3.8)	6 (7.1)	b
Hemorrhoids, n (%)	3 (10)	22 (15.9)	27 (20.8)	24 (28.6)	c
BBPS median [range]	9 [6–9]	9 [5–9]	9 [6–9]	9 [5–9]	NS
Medication usage					
Acetylsalicylic acid, n (%)	2 (6.7)	9 (6.5)	20 (15.4)	6 (7.1)	d
Carbasalate calcium, n (%)	3 (10)	22 (15.9)	12 (9.2)	14 (16.7)	NS
Clopidogrel, n (%)	2 (6.7)	0 (0)	3 (2.3)	2 (2.4)	NS
VKA, n (%)	0 (0)	3 (2.2)	8 (6.2)	1 (1.2)	NS
DOAC, n (%)	0 (0)	4 (2.9)	5 (3.8)	2 (2.4)	NS
NSAID, n (%)	0 (0)	4 (2.9)	5 (3.8)	12 (14.3)	e
Statin, n (%)	8 (26.7)	46 (33.3)	42 (32.3)	25 (29.8)	NS
ACE inhibitor, n (%)	8 (26.7)	43 (31.2)	31 (22.3)	18 (21.4)	NS
Bisphosphonate, n (%)	0 (0)	2 (1.4)	0 (0)	0 (0)	NS
Metformin, n (%)	4 (13.3)	7 (5.1)	10 (7.7)	6 (7.1)	NS
Insulin, n (%)	1 (3.3)	2 (1.4)	4 (3.1)	3 (3.6)	NS
Medical history					
Hypertension, n (%)	13 (43.3)	75 (54.3)	59 (45.4)	34 (40.5)	f
Diabetes, n (%)	5 (16.7)	10 (7.2)	16 (12.3)	11 (13.1)	NS
COPD, n (%)	2 (6.7)	16 (11.6)	10 (7.7)	7 (8.3)	NS
Cerebrovascular events, n (%)	9 (30)	31 (22.5)	33 (25.4)	20 (23.8)	NS
Hypercholesterolemia, n (%)	2 (6.7)	12 (8.7)	15 (11.5)	9 (10.7)	NS

AA, advanced adenoma; ACE, angiotensin converting enzyme; BBPS, Boston Bowel Preparation Scale; COPD, chronic obstructive pulmonary disease; CRC, colorectal cancer; DOAC, direct oral anticoagulant; NAA, nonadvanced adenoma; NS, not significant; NSAID, nonsteroidal antiinflammatory drug; VKA, vitamin K antagonist.

a CRC vs AA (*P* = 0.017), CRC vs NAA (*P* = 0.004), CRC vs controls (*P* = 0.027).

b AA vs controls (*P* = 0.008).

c CRC vs controls (*P* = 0.040), AA vs controls (*P* = 0.024).

d AA vs NAA (*P* = 0.020).

e CRC vs controls (*P* = 0.029), AA vs controls (*P* = 0.001), NAA vs controls (*P* = 0.006).

f AA vs controls (*P* = 0.045).

### Breath data analysis

#### Model 1: discriminating CRC vs negative controls.

**Figure 4.**
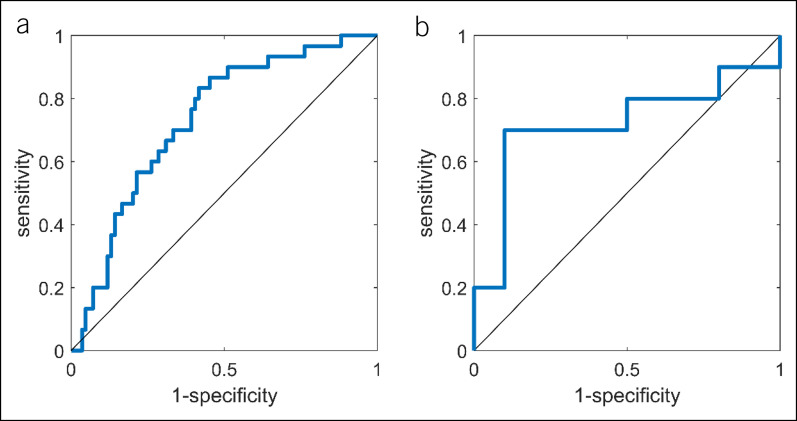
(**a**) The obtained ROC curve when comparing colorectal cancer with negative controls using the Isolation Forest procedure. An AUC ROC, sensitivity, and specificity of 0.7313, 67.3%, and 70% were obtained, respectively. (**b**) The obtained ROC curve for the independent test set of the Random Forest model comparing advanced adenoma cases with control cases using 10 discriminatory volatile organic compounds. The sensitivity and specificity were found to be 70% and 90%, respectively. AUC ROC = 0.727, AUC precision-recall = 0.662. AUC, area under the curve; ROC, receiver operating characteristic.

Model 1 distinguished CRC from negative controls using Isolation Forest with leave-one-out cross validation. The ROC curve of this model is shown in Figure 4a with an area under the curve ROC of 0.73. The obtained sensitivity and specificity were 67.3% and 70%, respectively.

#### Model 2: discriminating AA vs negative controls and CRC plus AA vs negative controls.

**Figure 5.**
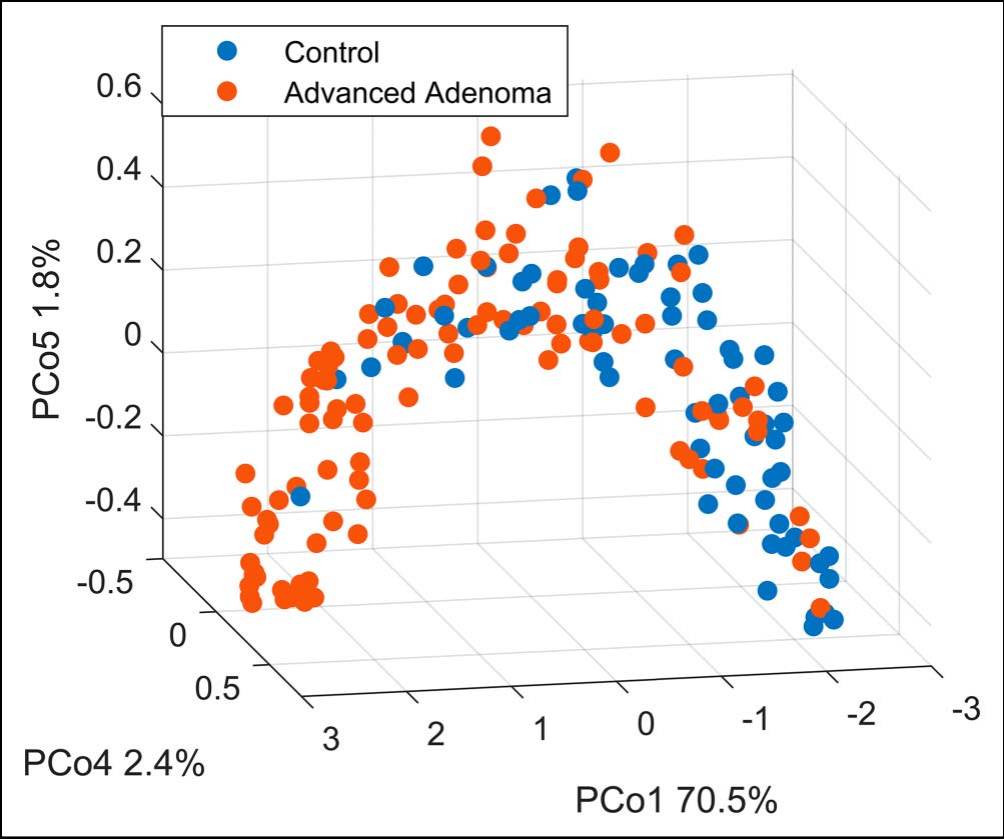
Principal Coordinate Analysis score plot on a proximity matrix obtained from the RF model and subsequently transformed using unsupervised RF built as described under the Methods section. The model was based on 10 selected volatile organic compounds. Every point belongs to a single breath fingerprint (red: advanced adenoma cases; blue: control cases). The separation is observed on Principle Component 1, explaining 70.5% of the variance. RF, Random Forest.

The final RF model built on the breath training data containing AA and negative controls identified a set of 10 discriminatory VOCs that yielded a sensitivity and specificity of 79% and 70%, respectively, for the internal out-of-bag validation set. Subsequently, when testing on the independent test set (n = 20), a sensitivity and specificity of 70% and 90%, respectively, were obtained. The PPV for this sample set increased from 63% based on FIT only to 87.5% based on FIT and breath combined. The ROC curve for the independent test set is shown in Figure 4b. In Figure 5, the separation between both groups is visualized. Furthermore, CRC could be discriminated from controls with a sensitivity and specificity of 80% and 70%, respectively, using the 5 most important VOCs from this model. In addition, CRC and AA combined as 1 group could be discriminated from controls with a sensitivity and specificity of 77% and 70%, respectively. ROC curves for these models and of the internal training set of Model 2 are reported in the supplementary material (see Supplementary Figures 1 and 2, Supplementary Digital Content 3, http://links.lww.com/CTG/A852).

**Figure 6.**
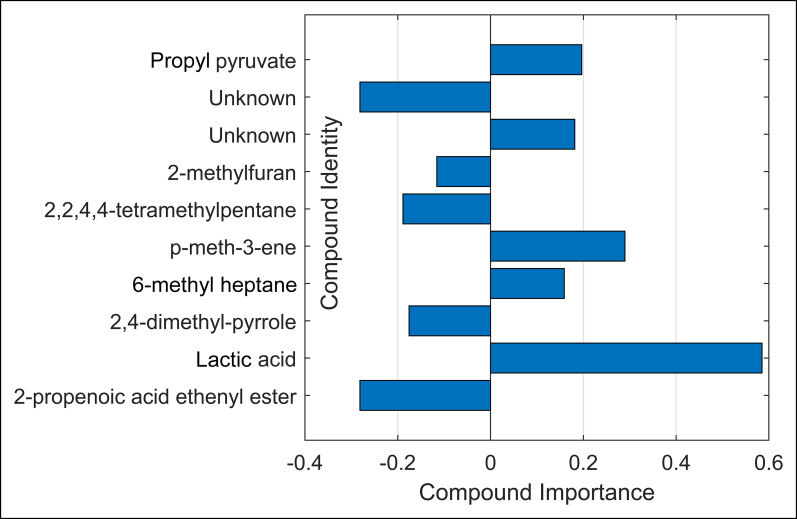
Importance of the 10 selected and identified volatile organic compounds. Negative values indicate higher concentrations in control samples; positive values indicate higher relative concentrations in advanced adenoma cases.

The set of 10 discriminatory VOCs were identified as 2-propenoic acid ethenyl ester; lactic acid; 2,4-dimethyl-pyrrole; p-menth-3-ene; 6-methyl heptane; 2,2,4,4-tetramethylpentane; 2-methylfuran; propyl pyruvate; and 2 unknown molecules because of low compound abundance. Of these, 2-propenoic acid ethenyl ester; lactic acid; 2,4-dimethyl-pyrrole; p-menth-3-ene; and 1 unknown molecule were most predictive for CRC and CRC and AA combined when compared with controls. The importance per compound is shown in Figure 6. Compounds with positive importance had higher concentrations in the AA group as compared with controls. Similarly, compounds with negative importance had lower concentrations in the AA group.

#### Model 3: discriminating AA vs NAA vs negative controls.

**Figure 7.**
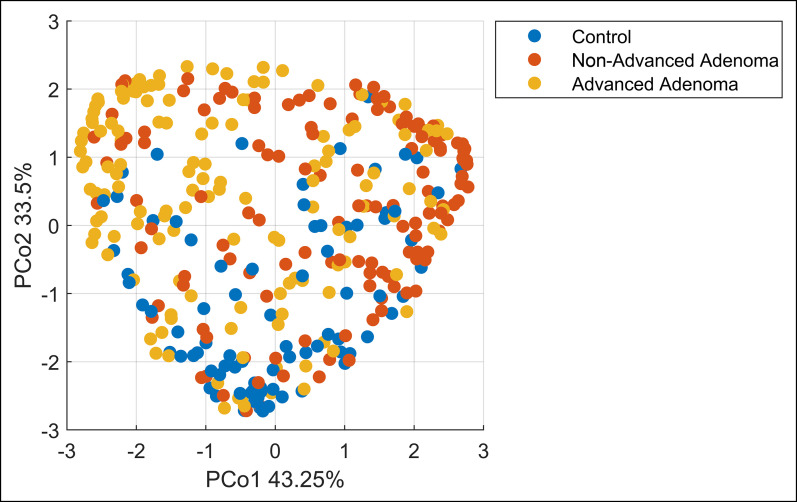
Principal Coordinate Analysis score plot on a proximity matrix obtained from the RF model built and subsequently transformed using unsupervised RF. The model was based on the midlevel fusion of Model 1 (advanced adenoma vs control) and the hierarchical subsequent models A and B. Every point belongs to a single breath chromatogram (blue: control cases; red: nonadvanced adenoma cases; yellow: advanced adenoma cases). The separation is observed on Principle Coordinates 1 and 2 together, explaining 76.75% of the variance. RF, Random Forest.

In Model 3, the hierarchical models A and B together discriminated (Model A) AA vs rest and (Model B) NAA and negative controls with a sensitivity and specificity of 72% and 65% using 12 VOCs for Model A and 75% and 78% using 13 VOCs for Model B, respectively. By midlevel fusion, the subsequently calculated PCoA scores for these models were combined with those of Model 2 and the final predictions were calculated. In Figure 7, the PCoA visualization of the final proximities is presented, showing a separation between the 3 classes and the potential to additionally separate NAA. The overall procedure resulted in an overall weighted accuracy of 54%, where an accuracy of 33% indicates random performance.

Although the performance of the model was validated using the internal validation sets, the results could not be verified in independent test sets. Because the Model was not independently validated, we recognize that the results in model 3 may be premature, and therefore, chemical identification of the discriminating components has not been performed.

## DISCUSSION

In this multicenter prospective study, the feasibility of exhaled breath analysis to differentiate between CRC, AA, NAA, and negative controls in a FIT-positive CRC screening population was assessed. CRC could be distinguished from negative controls with a sensitivity and specificity of 67.3% and 70%, respectively. In addition, AA could be discriminated from negative controls based on 10 discriminatory VOCs with a sensitivity and specificity of 79% and 70%, respectively. The combination of exhaled breath analysis with FIT led to an enhanced PPV of 82% as compared with 63% if only a FIT would have been applied. Interestingly, using the 5 most important VOCs specified by Model 2, both CRC as well as CRC and AA combined could be discriminated against controls with sensitivities of 80% and 77%, respectively, and a specificity of 70% for both. Finally, the feasibility of additionally discriminating NAA in a 3-class classification model was shown. The clinical application of breath-based analysis is 2-fold. First, exhaled breath analysis may be used in combination with FIT in a 2-step procedure to stratify patients at risk of CRC or AAs (i.e*.,* colonoscopy indicated). Second, breath-based analysis has the potential to replace FIT as a screening test that considers not only CRC but also clinically relevant AAs. For both approaches, further research and validation steps are required.

Several earlier studies have analyzed exhaled VOCs for the detection of CRC, but only few studied its potential for the detection of adenomas, in particular AA (16–21). Recently, Altomare et al found 14 discriminatory VOCs that could distinguish patients with CRC from noncancer controls. However, CRC cases were mainly of an advanced stage and no adenomas were included in the study (21). Similarly, Markar et al published a study in which 7 VOCs were found to be associated with CRC. They successfully discriminated patients with CRC and other controls groups, but only a small number of undefined adenomas (n = 7) were included (20).

Eight of 10 selected VOCs discriminating AA from negative controls were putatively identified in this study. Of these, 2-propenoic acid ethenyl ester; lactic acid; propyl pyruvate; and 2,4-dimethyl-pyrrole can be linked to changes in the metabolic microenvironment of the colon. For instance, hypoxic tumor cells have a high rate of aerobic glycolysis (Warburg effect), consume more glucose, and secrete large amounts of lactic acid as a waste product in their microenvironment (36). Indeed, a relative enrichment of lactic acid has been previously reported in studies comparing CRC vs controls (36–40). The enrichment of propyl pyruvate can be explained by similar mechanisms because its derivative pyruvate is a precursor of lactate (41,42). Although 2,4-dimethyl pyrrole could not directly be interpreted in light of CRC, its unsubstituted version pyrrole was observed in abnormal concentrations in fecal samples in ulcerative colitis and Crohn's disease. Similarly, 2-propenoic acid ethenyl ester could not directly be interpreted, but its derivative propionate has repeatedly been found in lower concentrations in CRC cases, which is in line with our results (43). The formation of 6-methyl heptane and p-3-menthene could be linked to oxidative stress, a well-known phenomenon in neoplastic disease (44). The origin of 2 VOCs remained ambiguous: 2,2,4,4-tetramethylpentane and 2-methylfuran. The former could be related to microbial activity and dysbiosis (45–47). 2-Methylfuran may be related to oxidative stress, smoking status, or coffee consumption (48,49).

This study had some limitations. First, limited data on smoking and alcohol usage were recorded during the prescreening intake. This may have resulted in confounding effects because 2-methylfuran is also related to smoking. Because none of the identified VOCs related to alcohol usage, its influence on the results is expected to be minimal. Dietary habits were not part of the data collection because the ideal diagnostic tool should be sufficiently robust to be applied in the general population, irrespective of various external factors, including dietary habits (50). Second, the CRC group was underpowered to reliably find biomarkers. However, CRC could be detected using VOCs found in Model 2 suggesting that CRC and AA are biologically closely related. Improved predictions are expected when more specific CRC markers are added in the model, for which larger study populations are required.

Future perspectives include the validation of our results in a larger cohort, which will additionally allow detection of CRC-specific biomarkers. Improved results can be expected because breath sampling methods have significantly been optimized over recent years. In addition, exhaled breath analysis should be tested in a FIT-naïve population as well, to verify the applicability of breath analysis as a standalone screening test.

A set of 10 discriminatory VOCs was identified that could detect AA when compared with controls and was related to the microenvironment of the colon. Furthermore, the feasibility of exhaled breath analysis to successfully detect CRC in a FIT-positive population was demonstrated. Further research is needed to validate these results, but this study has shown the potential of breath-based analysis to improve detection rates of both CRC and AAs, either in combination with FIT or as a standalone screening test.

## CONFLICTS OF INTEREST

**Guarantor of the article:** Agnieszka Smolinska, PhD.

**Specific author contributions:** H.C. and R.v.V. contributed equally and share cofirst authorship. H.C. obtained ethical approval, conducted the study, collected samples, and wrote the manuscript. R.v.V. chemically and statistically analyzed obtained samples and wrote the manuscript. D.P. was involved in patient sampling and chemically analyzed samples. L.M. and J.S. were involved in sample collection and database management. J.D. was involved in study design. D.J., A.M., F.S., Z.M., and A.S. were involved in study design, data collection, data interpretation, and constructive review of the manuscript. All authors had access to the study data and have reviewed and approved the final manuscript.

**Potential competing interests:** A.S. is assistant professor at Maastricht University and is an advisor at Owlstone Medical (Cambridge, UK), a breath-based medical company aiming for the noninvasive detection of diseases. The remaining authors declare that the research was conducted in the absence of any commercial or financial relationships that could be construed as a potential conflict of interest.

**Financial support:** The present study was supported by the Airborne Biomarkers for Colorectal Cancer project within the program ERA-NET: Transscan-2, Joint Translational Call for Proposals 2016 on: “minimally and noninvasive methods for early detection and/or progression of cancer,” transscan-067, KE, No. ERA-NET TRANSSCAN/02.2018. A project funded by the European Commission under the EU framework Horizon2020. The funding source had no role in study design, data collection and analysis, preparation of the manuscript, or decision to publish.

**IRB approval statement:** The study was approved by the Medical Ethics Research Committee of Maastricht University Medical Center+ (METC No. 16-4-103.1/ab).

**Study Highlights**

**WHAT IS KNOWN**

✓ Colorectal cancer can be prevented if precursor lesions are identified and removed endoscopically.
✓ Fecal immunochemical tests for hemoglobin for bowel cancer screening programs suffer from high numbers of false-positives and are insensitive for advanced adenomas.
✓ Exhaled volatile organic compounds have been studied for colorectal cancer, but data on (advanced) adenomas are limited.

**WHAT IS NEW HERE**

✓ Breath-based analysis has the potential to be used as a screening test for both colorectal cancer and clinically relevant advanced adenomas.

## Supplementary Material
