# Supplementary material for: Detecting Colorectal Adenomas and Cancer Using Volatile Organic Compounds in Exhaled Breath: A Proof-of-Principle Study to Improve Screening
Source: Clin Transl Gastroenterol. 2022 Aug 18;13(11):e00518. doi: 10.14309/ctg.0000000000000518 (PMC10476860; doi:10.14309/ctg.0000000000000518)
Supplement: Supplementary file 1 [file ct9-13-e00518-s001.docx]

**Supplemental Digital Content 3**

**Supplementary Figure 1**: PR (**A**) and ROC curve (**B**) for OOB internal training set of the RF model comparing AA cases to control cases using 10 discriminatory VOCs. The sensitivity and specificity were found to be 79% and 70%. AUC ROC=0.798, AUC PR=0.845. **C**: PR curve for results obtained using the independent test. The sensitivity and specificity were found to be 70% and 90%. AUC PR 0·662.
PR: precision recall, ROC: receiver operating characteristic, OOB: Out of Bag, RF: Random Forest, AA: advanced adenoma, VOC: volatile organic compounds, AUC: area under the curve.

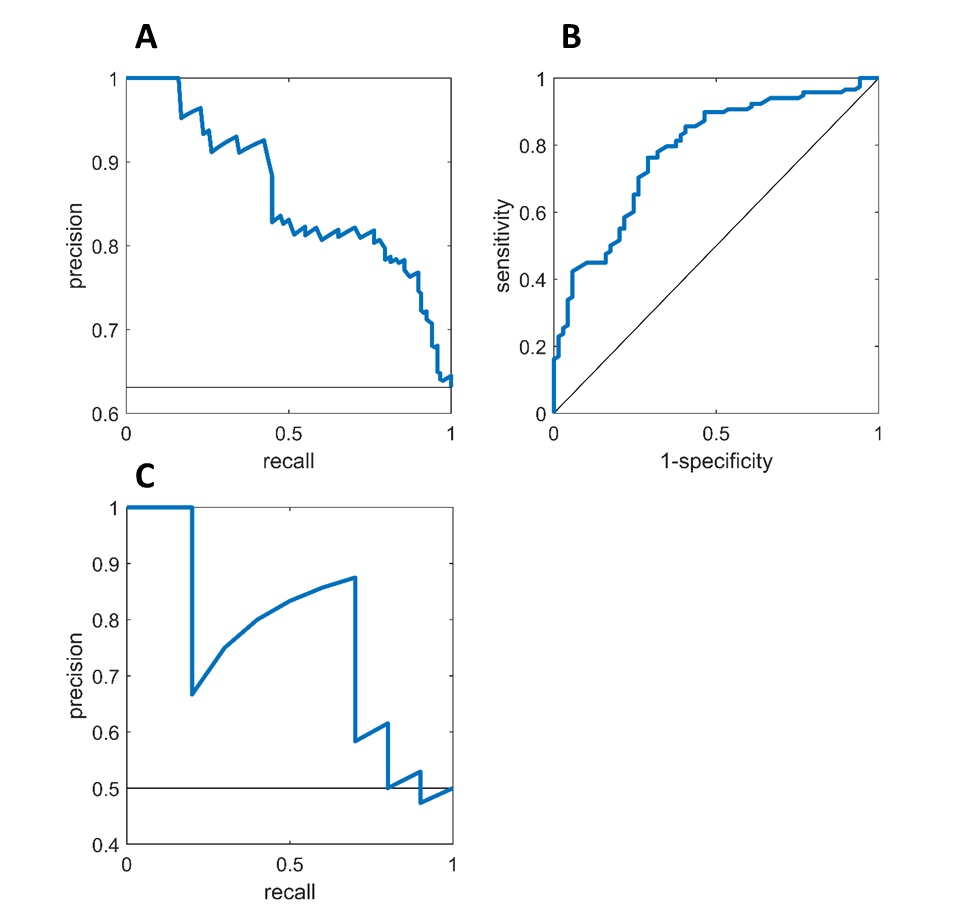


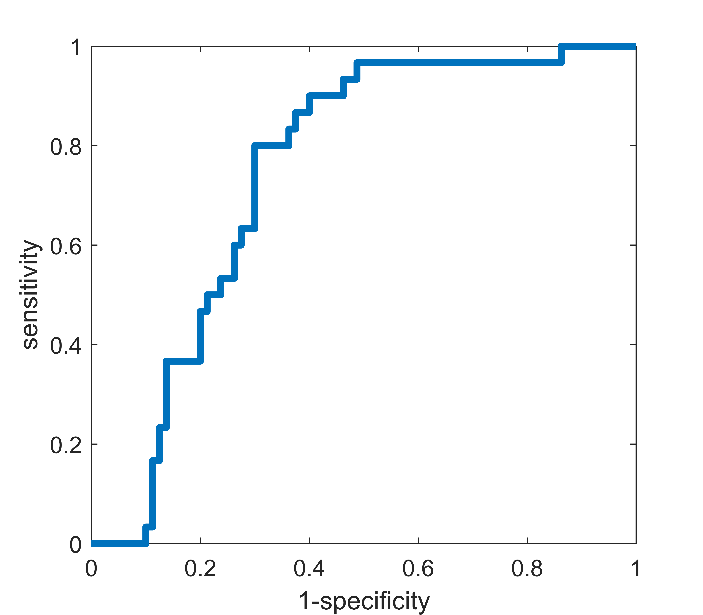

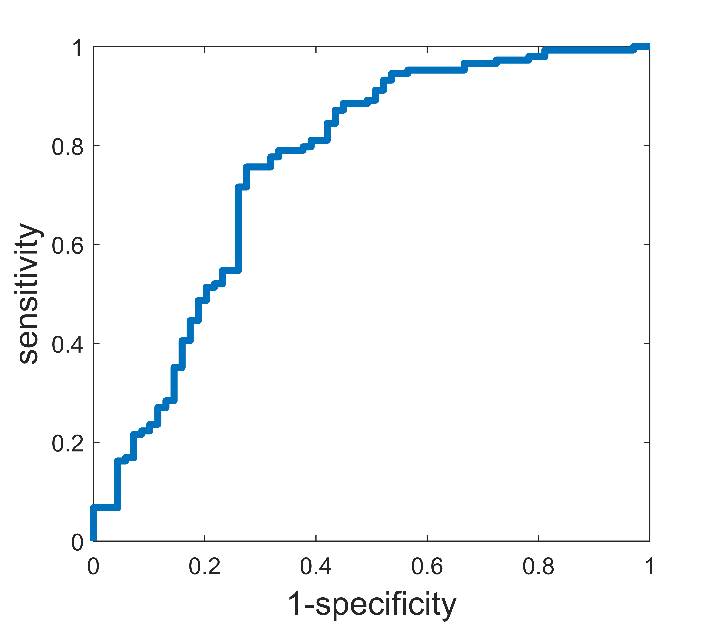
**A B**

**Supplementary Figure 2A:** The obtained ROC curve for the discrimination of CRC versus control using the five most important features from *Model* 2 (*i.e.* created to compare AA cases to control cases). The sensitivity and specificity were 80% and 70%, respectively. AUC ROC=0.762. **B:** The obtained ROC curve for the discrimination of CRC and AA combined versus control using the five most important features from *Model* 2 (*i.e.* created to compare AA cases to control cases). The sensitivity and specificity were 77% and 70%, respectively. AUC ROC=0.750.
