# Supplementary material for: Detecting Colorectal Adenomas and Cancer Using Volatile Organic Compounds in Exhaled Breath: A Proof-of-Principle Study to Improve Screening
Source: Clin Transl Gastroenterol. 2022 Aug 18;13(11):e00518. doi: 10.14309/ctg.0000000000000518 (PMC10476860; doi:10.14309/ctg.0000000000000518)
Supplement: Supplementary file 2 [file ct9-13-e00518-s002.docx]

**Supplemental Digital Content 1**

**Supplementary Table 1**: Technical summary of preprocessing exhaled breath GC-MS data. This consisted of noise removal via wavelets and baseline correction via P-splines.^1,2^ Next, chromatograms were aligned using Correlation Optimized Warping.^3^ Subsequently, Probabilistic Quotient Normalization together with peak picking based on retention times and mass spectra were applied to normalize and create the functional data matrix, respectively.^4^ Variables were transformed using zero-imputation and coxbox transformation.

| Pre-processing step | Methodology / Algorithm | Reference |
| --- | --- | --- |
| Noise Removal | *Wavelets* | 2 |
| Baseline Correction | *P-Splines* | 1 |
| Alignment | *Correlated Optimized Warping* | 3 |
| Normalization | *Probabilistic Quotient Normalization* | 4 |
| Peak Picking | *Min Max peak picking, using Total Ion Chromatogram* | 2 |
| Transformation | *Zero-imputation + coxbox transformation* | - |

^GC-MS: gas chromatography – mass spectrometry.^

**References**

1. Eilers PHC, Boelens HFM. Baseline Correction with Asymmetric Least Squares Smoothing. *Life Sci*. Published online 2005.

2. Smolinska A, Hauschild AC, Fijten RRR, Dallinga JW, Baumbach J, Van Schooten FJ. Current breathomics - A review on data pre-processing techniques and machine learning in metabolomics breath analysis. *J Breath Res*. 2014;8(2). doi:10.1088/1752-7155/8/2/027105

3. Tomasi G, Van Den Berg F, Andersson C. Correlation optimized warping and dynamic time warping as preprocessing methods for chromatographic data. *J Chemom*. Published online 2004. doi:10.1002/cem.859

4. Dieterle F, Ross A, Schlotterbeck G, Senn H. Probabilistic quotient normalization as robust method to account for dilution of complex biological mixtures. Application in1H NMR metabonomics. *Anal Chem*. Published online 2006. doi:10.1021/ac051632c
