# Supplementary material for: Detecting Colorectal Adenomas and Cancer Using Volatile Organic Compounds in Exhaled Breath: A Proof-of-Principle Study to Improve Screening
Source: Clin Transl Gastroenterol. 2022 Aug 18;13(11):e00518. doi: 10.14309/ctg.0000000000000518 (PMC10476860; doi:10.14309/ctg.0000000000000518)
Supplement: Supplementary file 3 [file ct9-13-e00518-s003.docx]

**Supplemental Digital Content 2**

**Breath Data Analysis Model 1 – Details**

One class classification models are trained using only one class and focus on similarities between samples, in contrast to more traditional discriminatory models that focus on differences between (typically two) classes. The procedure of isolation Forest (iF) makes use of isolation trees where, based on fully randomized splits of randomly selected features, data of the target class is split up into smaller aliquots. The pathlength (*i.e.* number of splits) after which a sample is fully isolated from the other samples is averaged over all trees and recorded as output measure, here isolation scores (IS), see Figure 2. Next, the IS of the non-target class are obtained and compared to those of the target class. Using this approach, the risk of reaching statistically biased and biologically irrelevant conclusions was minimized as isolation Forests are based on one class and circumvent the need for (potentially biased) feature selection. Note, this decrease in bias came at the cost of model interpretation, as isolation Forests function as *Black Boxes* and variable interpretation is not evident.

**
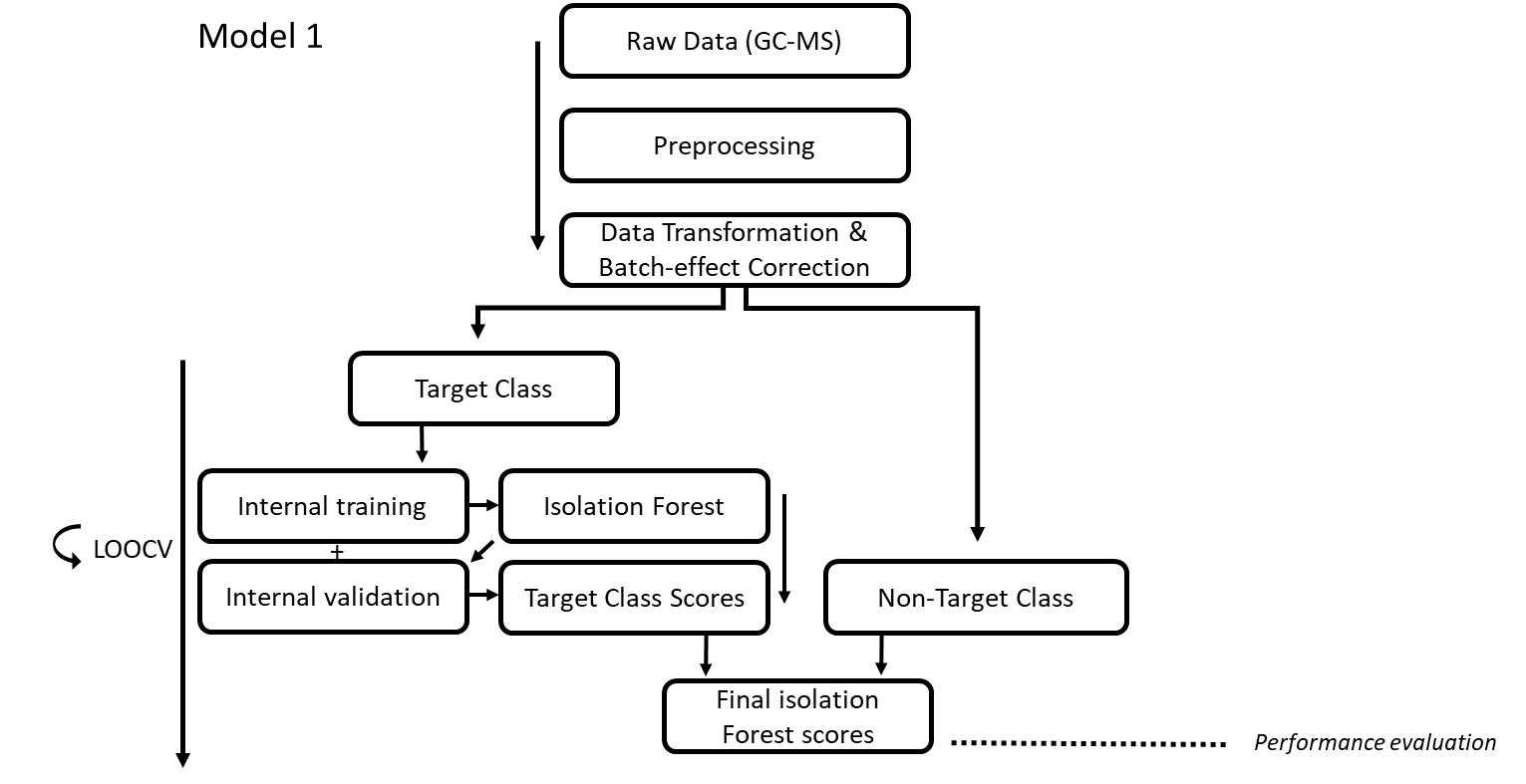

Figure 1:** Representation of the data-analytics that were applied in the current study to distinguish CRC from negative controls. In this study, COMBAT was applied as batch-effect correction technique. The entire isolation Forest procedure was performed twice, where training was performed on either one of the respective target groups. Upon obtaining the final isolation scores for each sample for both of the respective target classes (*i.e.* CRC and negative controls), Bayesian Statistics were applied to calculate the final likelihood ratio of belonging to the CRC class.


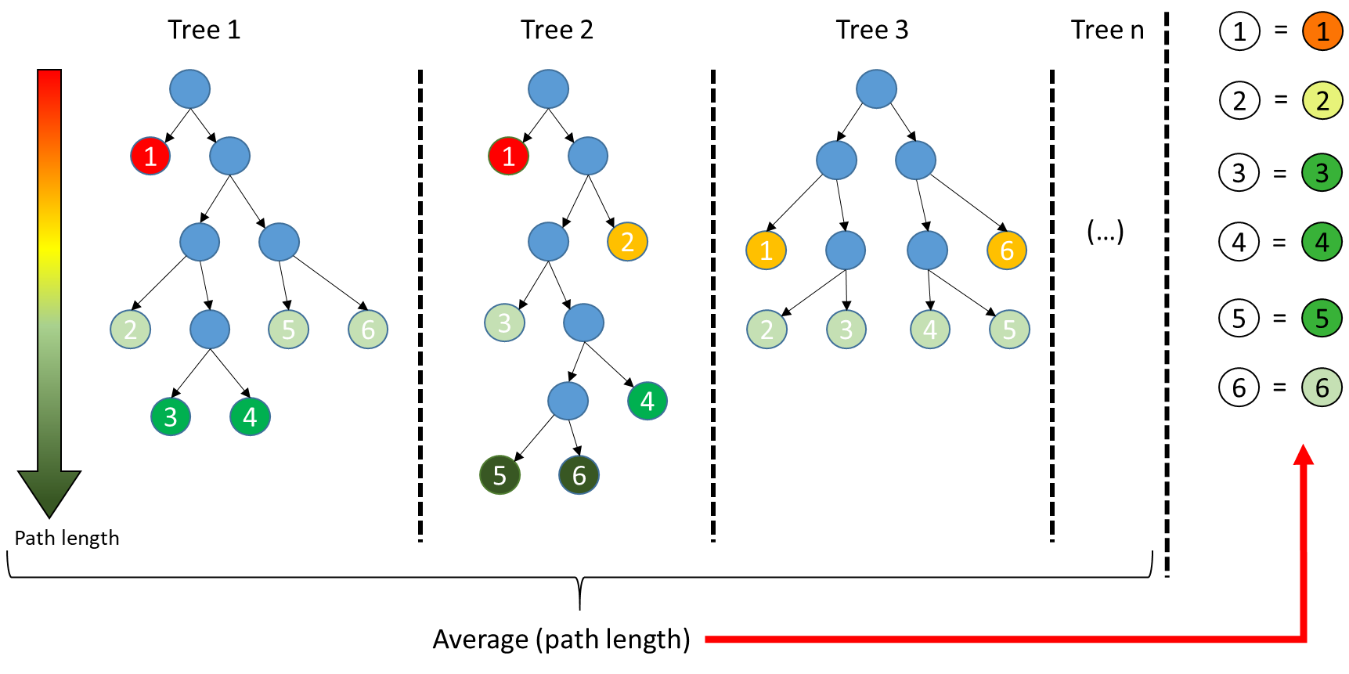


**Figure 2:** Isolation Forest explained. Isolation forest is based on decision making trees, here depicted as Tree 1, Tree 2 and Tree 3. In contrast to traditional decision making trees, isolation trees split data on fully randomized basis. Overall, a forest is grown consisting of n trees. Per tree the path length after which a sample is fully isolated is obtained and averaged over the entire forest. The obtained measure is the Isolation Score.

In this study isolation Forests were built using a *Leave One Out Cross Validation* (LOOCV) strategy. Two subsequent models were created: one based on colorectal cancer (CRC) cases and the other on negative controls. Having obtained the IS of the respective target class, the IS of the non-target classes were calculated using the trained iF. Next, the IS of both models were normalized using Kernel Density Estimators (KDE) with an optimized kernel bandwidth calculated with the *Maximum Likelihood Cross Validation* approach, again based on the respective target class.^1,2^ Lastly, Bayes Rule was applied to obtain the final likelihood of belonging to the CRC class. The results were assessed using receiver operating characteristic (ROC).

Although these models do not require the specification of the descriptive biomarkers, a large feature to sample ratio (*i.e.* large dimensionality) can reduce statistical power in isolation Forests, and therefore a more compact feature space was created by performing dimensionality reduction via Principal Coordinate Analysis (PCoA) on the sample proximities derived from One Class *repeated randomly cross validated* Unsupervised Random Forests (*i.e.* using *i*=100 iterations, with per iteration 75% and 25% of target class samples used for training and testing, respectively) and including Principal Coordinates (PCs) cumulatively explaining 80% of variance.^3^ Note, to make the Unsupervised Random Forest suitable for One Class Classification, the synthetic dataset was not created by randomly permuting the original variables, but instead by iteratively permuting homogeneously distributed variables. More details on Unsupervised Random Forest and PCoA are provided further below under Data Visualization.

**Data visualization**

To visualize the results PCoA was performed on the Out-Of-Bag (OOB) proximity matrix obtained from the RF models. Here, proximity can be considered as a similarity measure across samples. A PCoA model based on this proximity measure serves to visualize the overall distributions of distances between all samples. However, sampling points that lack distinguishable patterns (*e.g.* samples in overlapping regions and outliers) are predicted with high uncertainty and can blur such visualizations by adding uninformative sources of variation to the PCoA model. To obtain a better visualization of the effect of interest (*i.e.* the proximities in terms of predictions), the proximities were re-calculated using Unsupervised Random Forest before PCoA.^3^ During this procedure the algorithm searches for patterns in the data that distinguish it from identical but randomly permuted data, thereby focusing on samples predicted with high certainty. This approach allowed for a better visualization of the data structure and diminished the effect of samples predicted with low certainty.

**References**

1. Duin R. On the Choice of Smoothing Parameters for Parzen Estimators of Probability Density Functions. *IEEE Trans Comput*. 1976;C-25(11). doi:10.1109/TC.1976.1674577

2. Habbema, J. D. F., Hermans, J., and Van den Broek K. A stepwise discrimination analysis program using density estimation. *Proc Comput Stat*. Published online 1974:101-110.

3. Afanador NL, Smolinska A, Tran TN, Blanchet L. Unsupervised random forest: A tutorial with case studies. *J Chemom*. 2016;30(5):232-241. doi:10.1002/cem.2790
